# CREATIVE WRITING AS A TOOL FOR LESBIAN, GAY, BISEXUAL, TRANSGENDER, AND QUEER (LGBTQ+) HISTORY AND FUTURE MAKING

**DOI:** 10.1093/geroni/igac059.542

**Published:** 2022-12-20

**Authors:** Lujira Cooper, Austin Oswald

**Affiliations:** Services and Advocacy for GLBT Elders, New York, New York, United States; Goldsen Institute of the University of Washington , Seattle, Washington, United States

## Abstract

The shift toward embracing creative methods in qualitative research opens new possibilities for gerontologists and older adults to explore the nuances of aging and its affective undertones. This paper describes the process of facilitating a weekly creative writing group for lesbian, gay, bisexual, transgender, and queer (LGBTQ+) older adults and their subjective experiences. Various creative writing practices (e.g., poetry, fiction, short story, biography) facilitates the retelling of life events and reimaging of new futurities. Done in community, it creates opportunities for social connectedness, collective meaning making, and psychosocial and instrumental support. Creative writing is a useful method for describing the LGBTQ+ aging experience not fully realized in gerontology. Our findings demonstrate the utility of creative methods in describing and re-imagining LGBTQ+ aging histories and futures. We argue for more creative methods that re-present the complexities of LGBTQ+ aging.

